# Radiopaque Resorbable Inferior Vena Cava Filter Infused with Gold Nanoparticles

**DOI:** 10.1038/s41598-017-02508-3

**Published:** 2017-05-19

**Authors:** Li Tian, Patrick Lee, Burapol Singhana, Aaron Chen, Yang Qiao, Linfeng Lu, Jonathan O. Martinez, Ennio Tasciotti, Adam Melancon, Steven Huang, Mitch Eggers, Marites P. Melancon

**Affiliations:** 1 Department of Interventional Radiology, The University of Texas MD Anderson Cancer Center, Houston, TX 77030 USA; 2 College of Medicine, State University of New York Upstate Medical University, Syracuse, NY 13210 USA; 3 Innovative Nanomedicine Research Unit, Chulabhorn International College of Medicine, Thammasat University, Rangsit Campus, Pathum Thani 12120 Thailand; 4 McGovern Medical School, The University of Texas Health Science Center at Houston, Houston, 77030 TX USA; 5 Department of Chemical and Biomolecular Engineering, Rice University, 6100 Main Street, Houston, TX 77005 USA; 6 Center for Biomimetic Medicine, Houston Methodist Research Institute (HMRI), 6670 Bertner Ave., Houston, TX 77030 USA; 7 Department of Orthopedics and Sports Medicine, Houston Methodist Hospital, 6565, Fannin Street, Houston, TX 77030 USA; 8 Department of Radiation Physics, The University of Texas MD Anderson Cancer Center, Houston, TX 77030 USA; 9 Adient Medical, Pearland, TX 77584 USA; 10 Graduate School of Biomedical Sciences, University of Texas Health Science Center at Houston, Houston, TX 77030 USA

## Abstract

Failure to remove a retrievable inferior vena cava (IVC) filter can cause severe complications with high treatment costs. Polydioxanone (PPDO) has been shown to be a good candidate material for resorbable IVC filters. However, PPDO is radioluscent under conventional imaging modalities. Thus, the positioning and integrity of these PPDO filters cannot be monitored by computed tomography (CT) or x-ray. Here we report the development of radiopaque PPDO IVC filters based on gold nanoparticles (AuNPs). Commercially available PPDO sutures were infused with AuNPs. Scanning electron microscopy analysis confirmed the presence of AuNP on the surface of PPDO. Micro-CT and x-ray images of the AuNP-infused PPDO sutures showed significant signal enhancement compared to untreated PPDO sutures. Elemental analysis showed that gold loading exceeded 2000 ppm. Tensile strength and *in vitro* cytotoxicity showed no significant difference between AuNP-infused and untreated PPDO. In a 10-week stability study, neither the gold content nor the radiopacity of the infused PPDO sutures significantly changed in the first 6 weeks. The increased attenuation of AuNP-infused PPDO sutures indicates their major advantage as a radiopaque resorbable filter material, as the radiopacity allows monitoring of the position and integrity of the filter, thereby increasing its safety and efficacy.

## Introduction

Inferior vena cava (IVC) filters are currently used for prevention of venous thromboembolism (VTE), which includes pulmonary embolism and deep vein thrombosis, when anticoagulants are contraindicated, bleeding complications occur during antithrombotic treatment, or VTE recurs despite optimal anticoagulation^1, 2^. IVC filter use has grown rapidly in recent years with the advent of retrievable IVC filters, which mitigate some of the risks associated with permanent IVC filters^1, 2^. However, only 22% of the patients get their retrievable IVC filter retrieved due to poor patient follow-up and other technical difficulties^3^. Failure of IVC filter removal can lead to severe complications including death and permanent injury^4^. Therefore, there is a great need for a resorbable filter that could circumvent the need for removal while still providing immediate protection against life-threatening VTE.

The polymer polydioxanone (PPDO) has been shown to be a strong candidate for use in resorbable IVC filters. PPDO has favorable tensile strength retention as well as adequate absorption time compared with other polymers tested^5^. However, PPDO is not radiopaque, a substantial disadvantage during insertion and subsequent monitoring^6^. Thus, there is a potential benefit in loading PPDO with a contrast agent for use in IVC filters, both increasing safety and efficacy while ensuring proper placement. Because of its tendency to swell in organic solvents, PPDO can be loaded with various compounds such as ibuprofen, triclosan, 5-fluorouracil, and even siRNA^7–10^. This tendency to swell also provides a potential to infuse PPDO with a contrast agent to make it radiopaque. We have previously successfully infused PPDO with iodine-based contrast agents 4-iodobenzoyl chloride (IBC) and 2,3,5-triiodobenzoic acid (TIBA). PPDO infused with either of these contrast agents exhibited significantly greater Hounsfield units (HU) than untreated PPDO on micro-computed tomography (micro-CT), with no noticeable change in surface morphology^11, 12^. The PPDO infused with TIBA, furthermore, showed neither significant loss in tensile strength nor adverse changes in crystallinity or melting temperature, indicating that TIBA is more suitable than IBC for IVC filters^11, 12^.

However, small molecule iodine-based contrast agents have numerous shortcomings, such as short imaging times, the need for catheterization, occasional renal toxicity, and poor contrast in large patients^13^. Interest in iodine as a contrast agent has declined not only because of these shortcomings but also because of its moderate atomic number compared with other suitable elements and because of its low K-shell binding energy. The K-edge is a sudden increase in the linear mass x-ray absorption coefficient occurring at a characteristic energy just above the binding energy of the electrons in the K-shell of the atom. With its low K-shell binding energy, iodine has suboptimal x-ray absorption coefficients at 100–130 kVp, the most common x-ray tube potential range used by many clinical imaging scanners. Because of this, nanoparticles of higher atomic numbers, such as gold, provide several advantages over these widely used iodinated contrast agent solutions^14^. Among the most studied are gold nanoparticles (AuNPs); gold, having a higher atomic number (Z = 79) and K-edge value (80.7 keV) than iodine (Z = 53, K-edge 33.18 keV), provides superior absorption of x-rays, resulting in better contrast than iodine on CT^14, 15^.

It is not only these attributes that give AuNPs a high potential to replace iodine-based contrast agents^13, 16^. Liu *et al*. recently demonstrated that AuNPs have greater attenuation than traditional iodine-based contrast agents for targeted CT^15^. In addition, clearance of AuNPs from the body is slower than that of iodine-based contrast agents, allowing for longer imaging time^13^. Conjugation of AuNPs via insertion of various biochemical moieties onto the surface of the nanoparticle could potentially allow for targeted delivery to a specific tissue or organ for direct imaging^14^. Although still an area of active debate, AuNP cytotoxicity is believed to be relatively mild, making them biocompatible^17, 18^. Conner *et al*. studied the acute toxicity of AuNPs in human leukemia cells and showed that they had no detrimental effect on cellular function^19^.

We hypothesized that infusion of PPDO with AuNPs would produce a radiopaque, x-ray– and CT-visible material suitable for IVC filters. The objectives of the research were to examine the feasibility of infusing AuNPs into commercialized PPDO sutures to make them radiopaque and to determine whether this infusion would compromise the sutures’ mechanical strength. Our results show that the signal intensity of the AuNP-infused PPDO sutures on CT and x-ray was greater than that of PPDO sutures without AuNPs, without a significant difference in mechanical strength. Our findings suggest that PPDO infused with AuNPs could potentially produce a superior radiopaque resorbable IVC filter. Enhancing the radiopacity of the resorbable PPDO filter would be a crucial step forward from a procedural and patient perspective.

## Results

### Synthesis and Characterization of AuNPs

**Figure 1:**
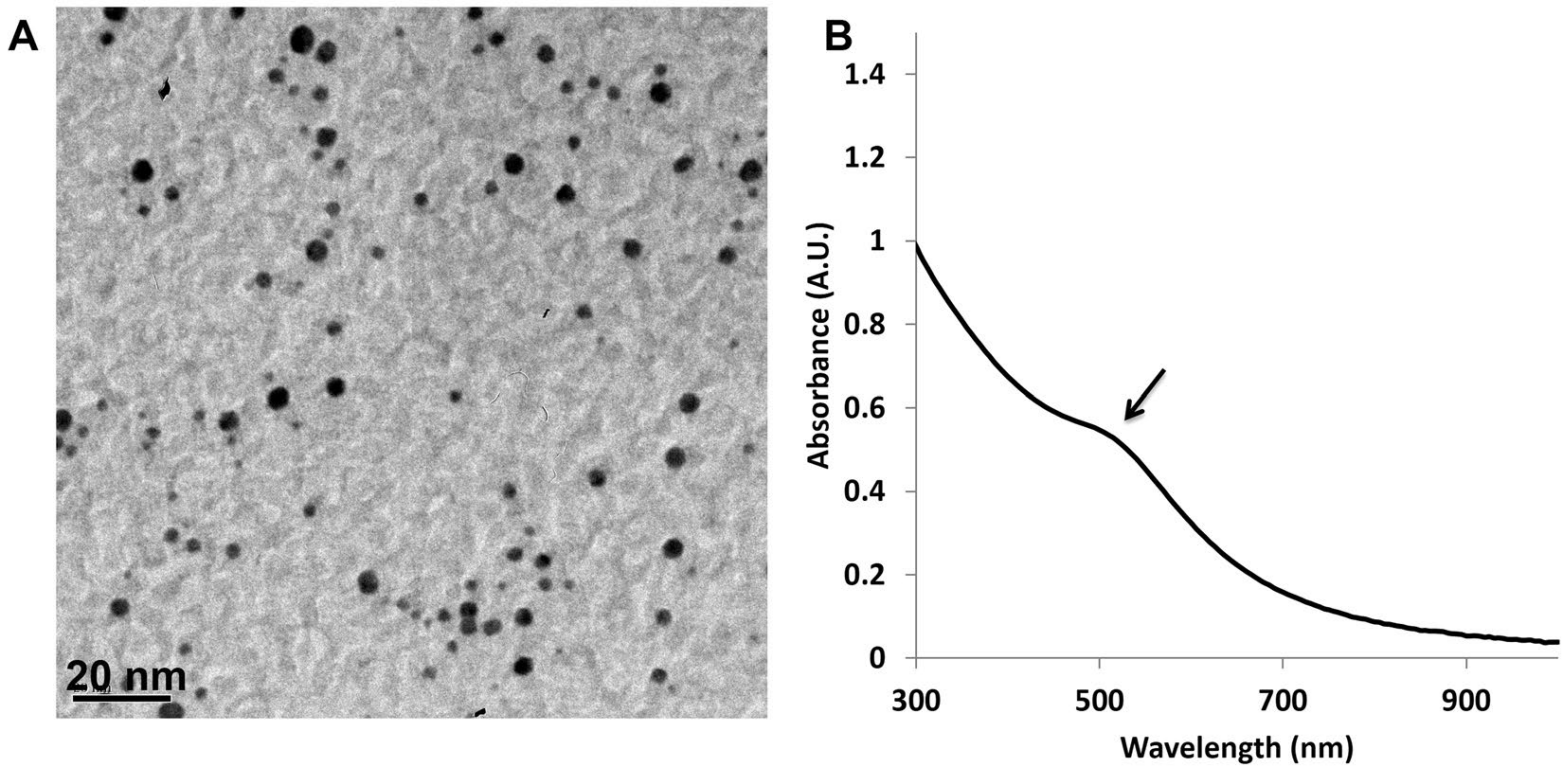
Hydrophobic 4-nm gold nanoparticles were successfully synthesized. TEM and UV-Vis spectrum of 4-nm AuNPs. The transmission electron microscopy (TEM) result (**A**) is consistent with the ~520 nm peak (shown by the arrow) on the UV-Vis spectrum (**B**) for the 4-nm AuNPs.

The successful synthesis of hydrophobic 4-nm AuNP was shown on both TEM and UV-Vis spectrophotometry (Fig. 1). Transmission electron microscopy (TEM) images showed that the synthesized AuNPs had an average size of approximately 4 nm. UV-Vis showed an absorbance peak at ∼520 nm, which coincided with the UV profile of the 4-nm AuNPs^20^.

### SEM Characterization, Radiopacity and Gold Content of AuNP-Infused PPDO Sutures

The soaking of the sutures in the AuNP-DCM or DCM caused the solution to turn violet from the dye. The control sutures turned pale and the AuNP-infused sutures obtained a visible metallic luster, after the violet dye was washed off.

**Figure 2:**
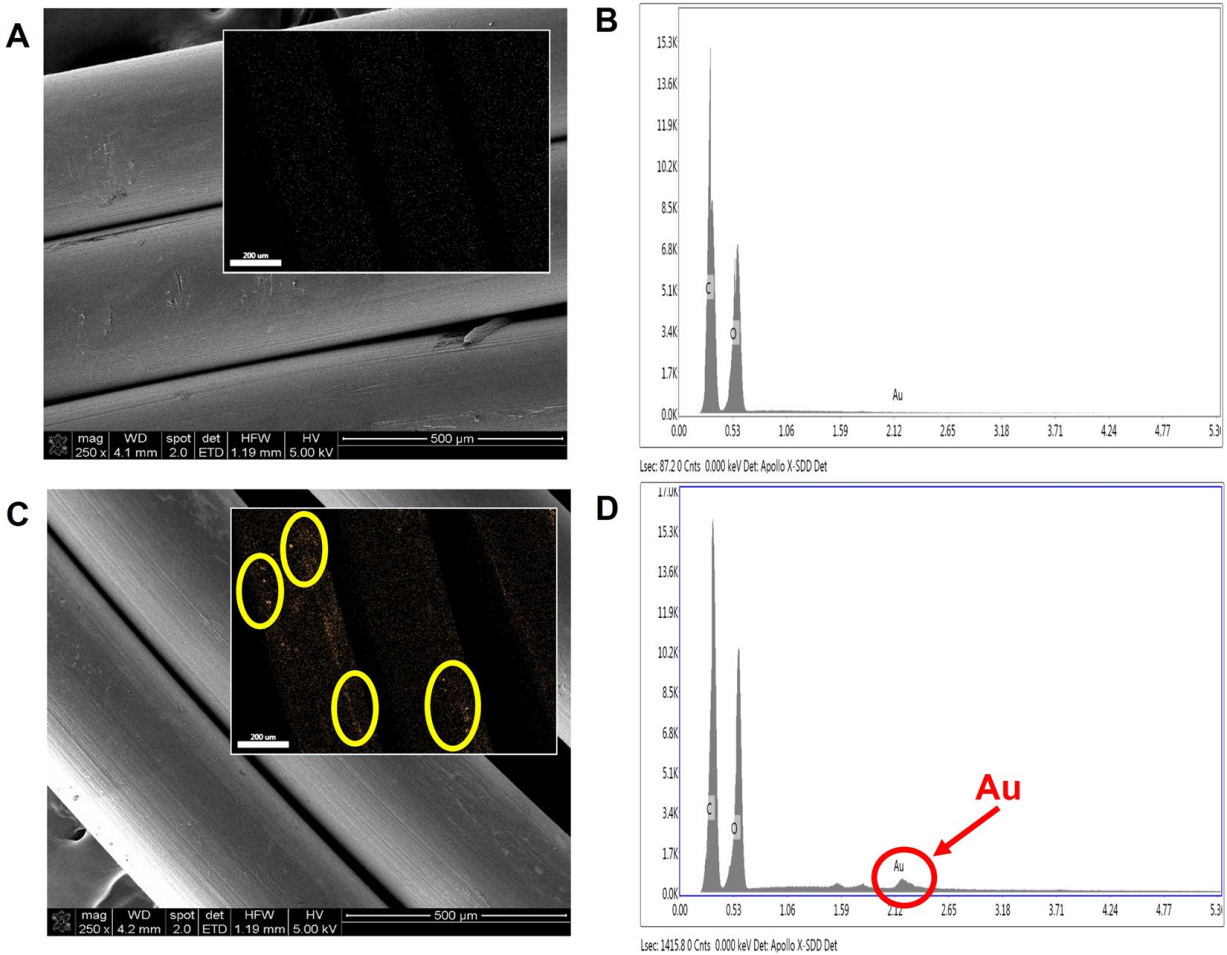
PPDO suture morphology was unchanged by gold nanoparticle infusion. (**A**) SEM of untreated PPDO suture (scale bar 500 μm). Inset shows the Au mapping from SEM-EDX (scale bar 200 nm). No Au signal was observed with the control. (**B**) SEM-EDX elemental analysis of untreated PPDO. No Au peak was observed. (**C**) SEM of AuNP-infused PPDO suture (scale bar 500 μm). Inset shows the Au mapping from SEM-EDX (scale bar 200 nm). Both AuNP clusters and individual AuNPs (pseudo-colored orange specs inside the yellow circles) were clearly visible on AuNP-PPDO but not on untreated PPDO. (**D**) SEM-EDX elemental analysis of untreated PPDO. A clear Au peak was observed.

Figure 2 shows Scanning Electron Microscopy coupled with Energy Dispersive X-ray (SEM-EDX) images of untreated and AuNP-infused PPDO sutures. No difference in morphology were observed on the surface of the sutures, but AuNPs were clearly visible on the AuNP-infused PPDO sutures (Fig. 2A and B). Element mapping by SEM-EDX is included as the insets of Fig. 2A and B. This demonstrates the presence of individual and clusters AuNP both on Au channel and the merged image of AuNP-PPDO. No AuNP was observed in untreated PPDO (yellow circles in the inset of Fig. 2B). Quantification in SEM-EDX also clearly showed the presence of Au peak on AuNP-infused PPDO sutures (1.14 ± 0.08% by weight), but not untreated PPDO sutures (0.19 ± 0.01% by weight).

**Figure 3:**
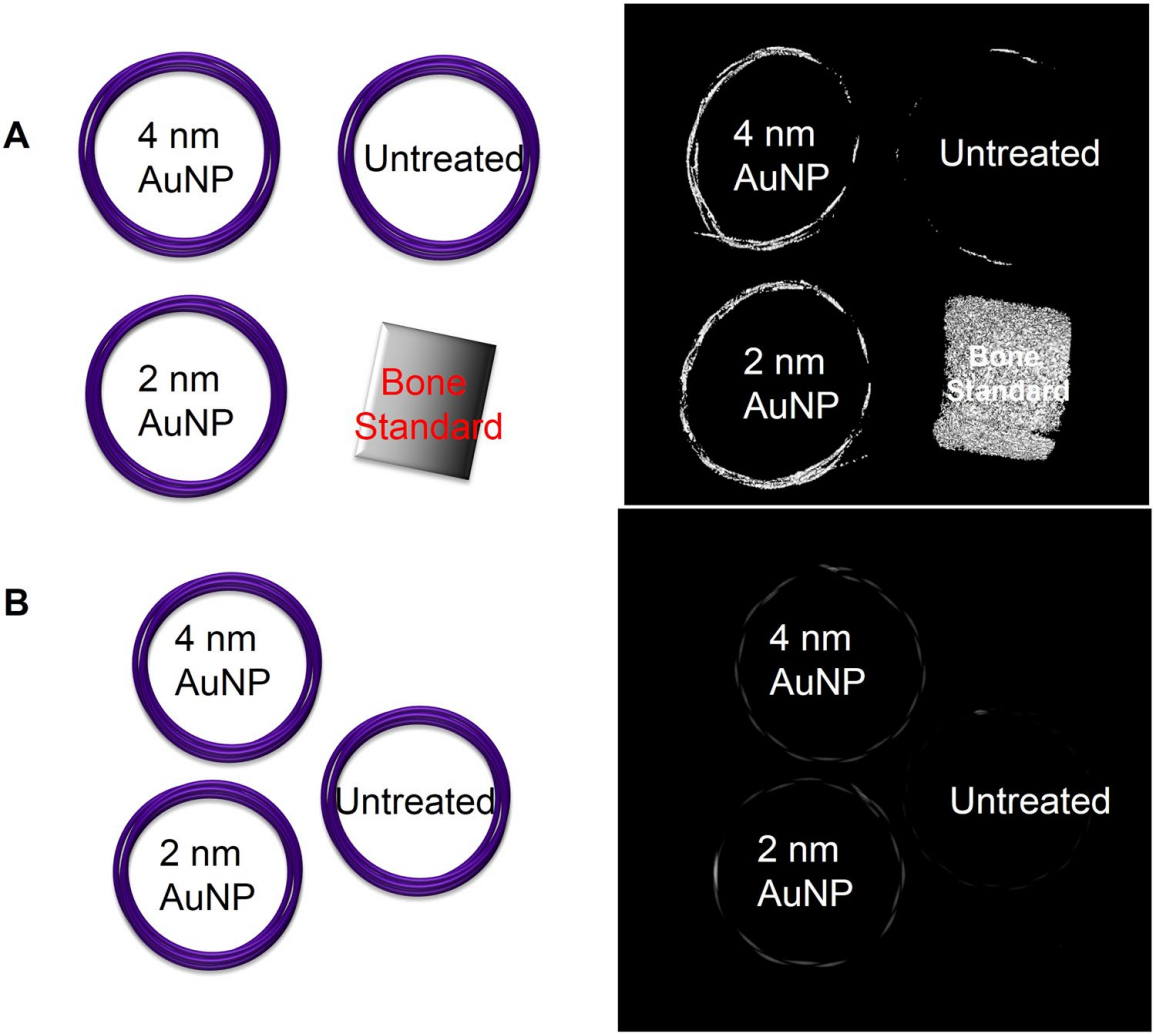
Infusion with gold nanoparticles increased radiopacity of PPDO sutures. Micro-CT (**A**) and x-ray (**B**) untreated showed higher attenuation of PPDO sutures infused with 2-nm or 4-nm AuNPs. The left panel shows the schematic arrangement of the PPDO sutures with bone standard.

The effects of particle size on gold loading and enhancement on micro-CT and x-ray imaging were determined by using 2- and 4-nm AuNPs. On ICP-OES, the amount of AuNP loaded into the PPDO sutures at an AuNP concentration of 5 mg/mL was 0.101 ± 0.035 ppm for the 4-nm AuNPs and 0.082 ± 0.012 ppm for the 2-nm AuNPs. These results show that there was no significant difference in the infusion efficiency between the two AuNP sizes (*t*-test, *p* = 0.40). These results were confirmed by micro-CT imaging and x-ray (Fig. 3). Unlike the untreated PPDO sutures, the AuNP-infused PPDO sutures are clearly visible on these images, and their intensity is comparable to that of bone. Both results suggest that infusion with AuNPs can enhance the radiopacity of the sutures, making them suitable for conventional imaging.

**Figure 4:**
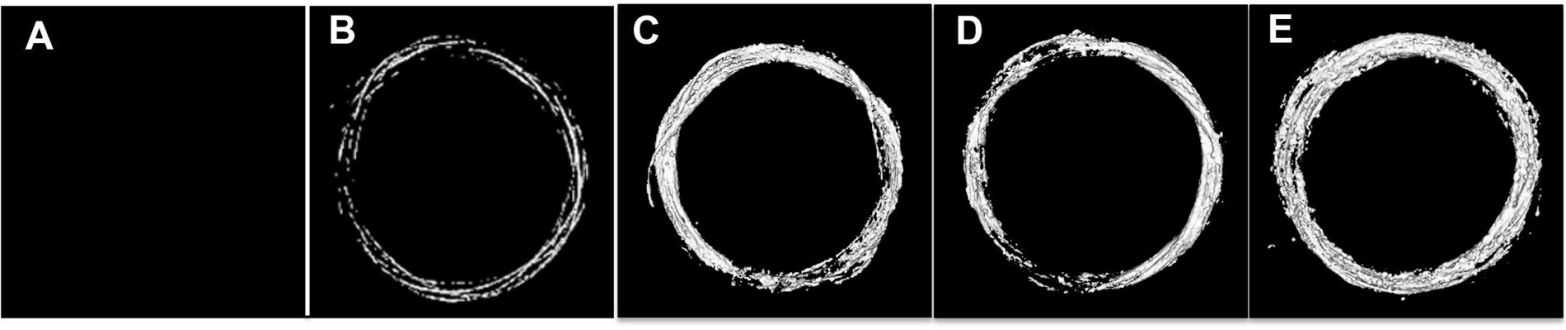
Greater concentration of gold nanoparticles in the infusion solution results in greater radiopacity. Untreated PPDO suture (**A**), iodine-infused PPDO suture (**B**) and PPDO sutures infused with a solution containing 50 mg/mL (**C**), 75 mg/mL (**D**), or 200 mg/mL (**E**) of AuNPs. Micro-CT attenuation increased with AuNP concentration.

We then evaluated the effects of different AuNP infusion solution concentrations (50, 75, and 200 mg/mL) on the loading efficiency using our synthesized 4-nm AuNP (Fig. 1). Micro-CT images showed greater attenuation for AuNP-infused sutures at different AuNP concentrations (Fig. 4C–E) as compared to untreated (Fig. 4A) and iodine-infused PPDO sutures (Fig. 4B). Infusing solutions containing 50 or 75 mg/mL of gold yielded gold loading in the PPDO sutures of 1637 ± 15.3 and 1702.9 ± 16.1 ppm, respectively, while the infusing solution containing 200 mg/mL gold yielded gold loading in the PPDO sutures of 971.1 ± 10.3 ppm.

### Mechanical Strength and Integrity of Infused PPDO Sutures

**Figure 5:**
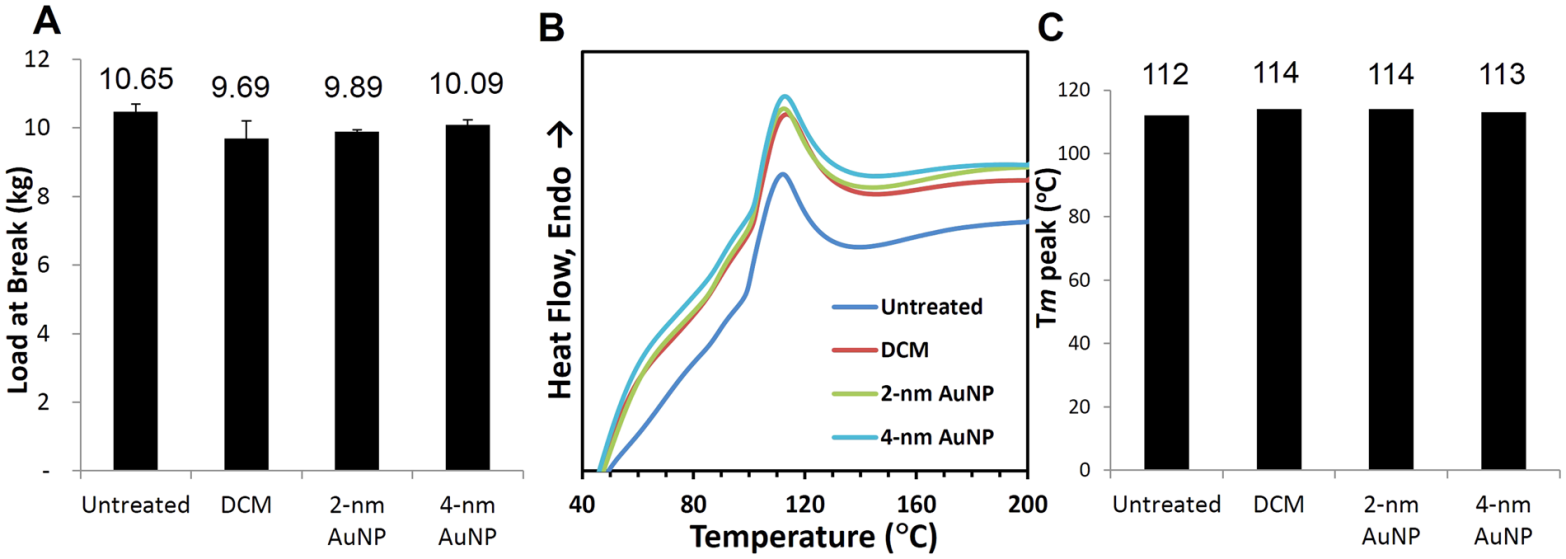
Infusion with gold nanoparticles had no significant effect on mechanical strength or integrity of PPDO sutures. The mechanical strength of the untreated and AuNP-infused PPDO sutures was tested by (**A**) the load-at-break method, (**B**) evaluating heat flux at various temperatures, and (**C**) melting temperature (T_*m*_) of each sample by differential scanning calorimetry. No significant differences were noted on any of these parameters. The load-at-break values did not differ significantly between the two groups (Kruskal−Wallis one-way ANOVA on ranks, *p* < 0.05). The heat reflux curves superimposed on each other, and all measured T_*m*_ values were around 112 °C.

The suture strength was tested by quantifying the load-at-break. PPDO infused with 2- or 4-nm AuNPs were tested, as were untreated sutures and sutures treated with DCM only. No significant difference in tensile strength was observed (Fig. 5A, Kruskal−Wallis one-way ANOVA on ranks, *p* < 0.05).

The integrity of the AuNP-infused and control sutures was evaluated by differential scanning calorimetry. The heat flux vs temperature curve is shown in Fig. 5B. The curves for all PPDO suture samples were superimposed on each other, suggesting that infusion of AuNPs did not alter suture integrity. Furthermore, there was no significant difference among the T_*m*_ peak values obtained from each PPDO suture (Fig. 5C).

### Cytotoxicity in RF24 and MRC5 Cells

**Figure 6:**
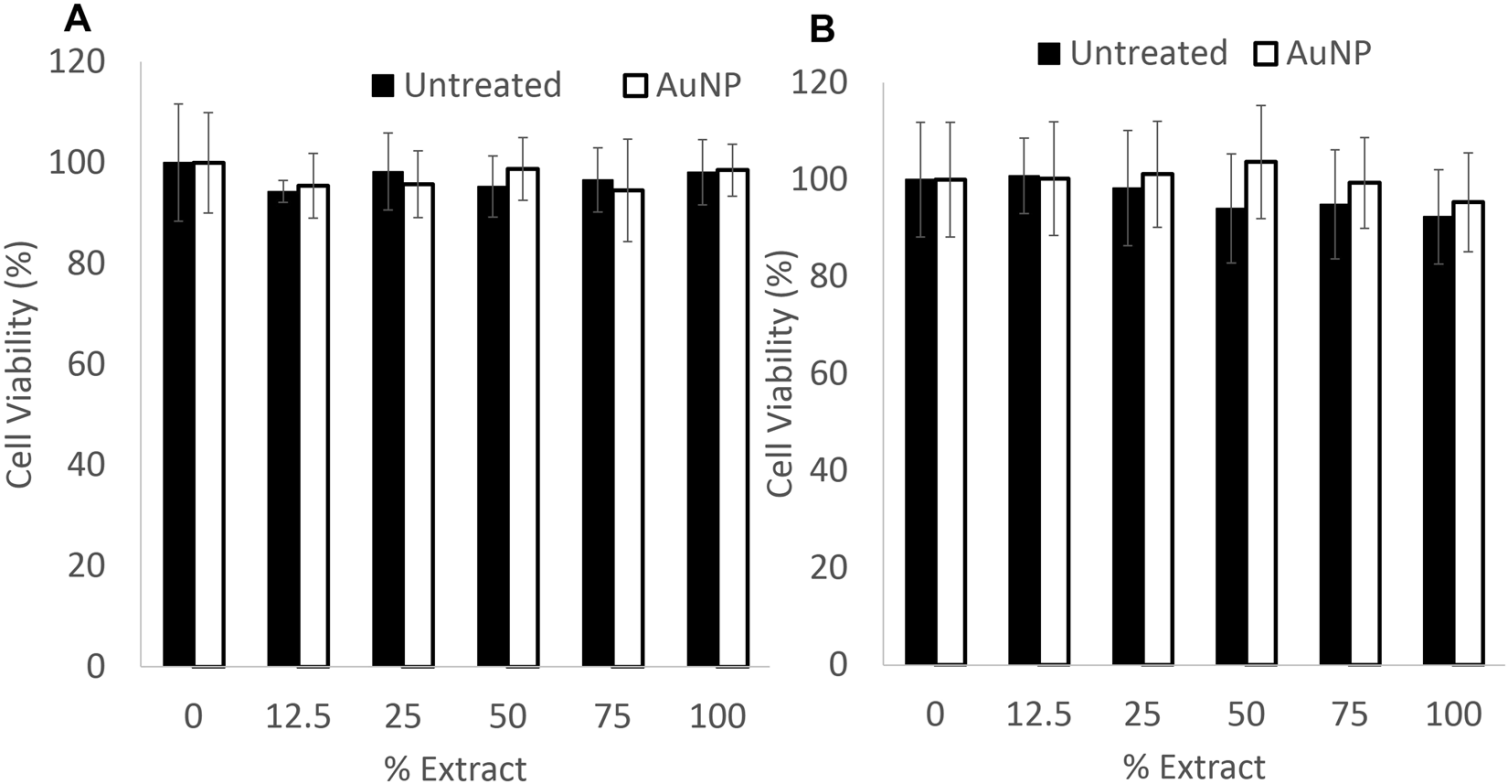
Infusion with gold nanoparticles did not increase cytotoxicity. Control and AuNP-infused PPDO sutures were subjected to extraction in MEM for RF24 (**A**) and EMEM for MRC5 (**B**) and diluted to various concentrations. The cytotoxicity of each concentration on RF24 and MRC5 cells was evaluated. No significant difference in cell viability was found between the treated groups and the control group (one-way ANOVA, *p* < 0.05).

The cytotoxicity of the AuNPs was tested to evaluate the biocompatibility of the AuNP-infused sutures. RF24 and MRC5 cells were treated with medium conditioned by AuNP-infused or control sutures (Fig. 6). There was no significant difference in cell viability among the treatment groups, or between the treatment groups and the control group (one-way ANOVA, *p* < 0.05).

### Long-term Radiopacity and Gold Content of Infused PPDO

**Figure 7:**
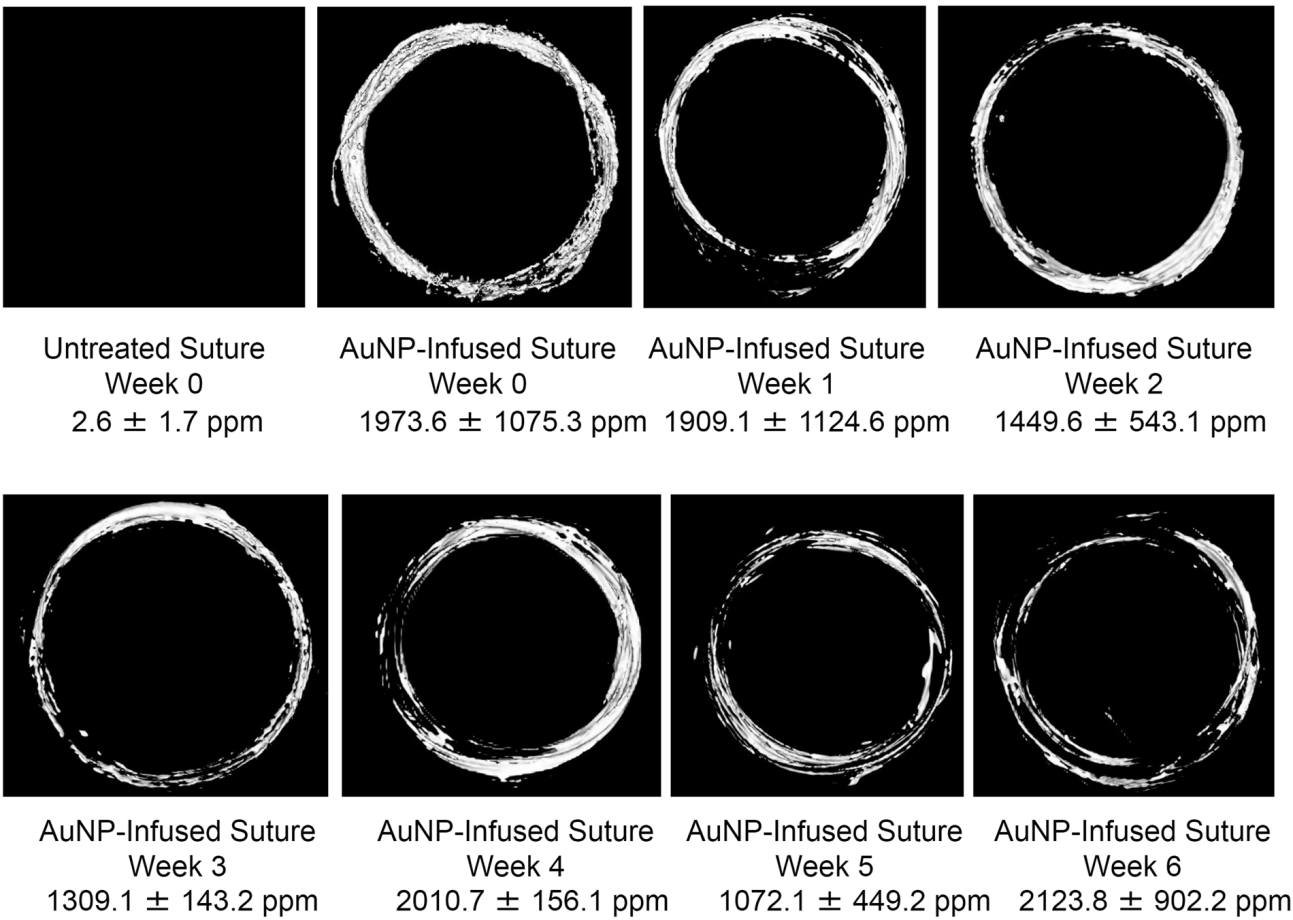
Long-term exposure to physiologic conditions did not affect radiopacity or gold content of gold nanoparticle–infused PPDO sutures. AuNP-infused PPDO sutures were suspended in PBS at 37 °C for up to 10 weeks. Three sutures were collected each week and imaged by micro-CT to determine radiopacity. Representative images over weeks 1–6 of the observation period are shown. The gold content was measured by ICP-OES, and the numeric result in ppm is listed under each image. All AuNP-infused PPDO sutures maintained radiopacity, and gold content did not decrease significantly during weeks 0–6 (one-way ANOVA, *p* = 0.778).

Changes in radiopacity and gold content of AuNP-infused and untreated PPDO sutures over a 10-week incubation period were evaluated by micro-CT and ICP-OES, respectively (Fig. 7). From week 7 on, both AuNP-infused PPDO sutures and untreated sutures started to lose substantial integrity and broke into pieces. No micro-CT images were taken from weeks 7–10. The gold contents of sutures from weeks 7–10 by ICP-OES were 1441.2 ± 840.2 ppm (week 7), 2128.8 ± 1270.4 ppm (week 8), 1716.1 ± 778.2 ppm (week 9), and 1856.3 ± 169.5 ppm (week 10). Micro-CT images showed consistently greater signal enhancement in the AuNP-infused PPDO sutures than in the untreated PPDO over weeks 1–6. The consistently greater radiopacity in AuNP-infused sutures was confirmed by ICP-OES results showing that the gold loading of the AuNP-infused sutures was consistently greater than that in the control sutures. For the AuNP-infused PPDO suture group, the differences in mean gold loading through the 10 weeks were not significant and were not great enough to exclude the possibility that the differences are due to random sampling variability (one-way ANOVA, *p* = 0.778).

## Discussion

Our results show that infusion of PPDO sutures with AuNPs significantly increased the polymer’s radiopacity on micro-CT and x-ray imaging without changing the integrity or altering the morphology of the suture. This radiopacity could allow monitoring of both position and integrity of a resorbable PPDO IVC filter over its typical dwell time, increasing its safety and efficacy as a medical device.

The fact that PPDO swells but does not dissolve in certain organic solvents, including DCM and chloroform^8^, allows PPDO sutures to be infused with small-molecule drugs^7, 8^ without significant change of their mechanical properties. On the basis of this finding, our laboratory has pursued the development of a radiopaque IVC filter device by infusing PPDO with iodine-based small molecule contrast agents IBC and TIBA^12^. However, iodine’s smaller atomic number and low K-shell binding energy of 33.18 keV resulted in suboptimal x-ray absorption. Gold, having a higher atomic number (Z = 79) and k-edge value (80.7 keV), provides superior absorption of x-rays, which results in better contrast upon CT^14^. Earlier studies showed that x-ray attenuation changes as the size of the gold particles infused is altered. For example, 4-nm AuNPs yielded a greater attenuation than Omnipaque, a commercially available iodine-based contrast agent, or of AuNPs of any larger size under the same molarity^21^. In the study presented here, the 4-nm AuNP was again shown to not only increase x-ray attenuation but also potentially allow infusion in greater amounts than larger nanoparticles.

Incorporation of AuNP within the PPDO after the infusion was confirmed by both SEM-EDX and ICP-OES. When two AuNP sizes were tested (2 and 4 nm), the particle size did not affect the final amount of gold loaded at the low concentration used (5 mg/mL). However, when the gold concentration of the infusing solution was increased to 50 or 75 mg/mL, the amount of gold loaded increased significantly. Since the loading of AuNPs into the sutures was driven by passive diffusion, a higher concentration in the infusing solution would produce a higher gradient and thus higher loading. But increasing the AuNP concentration further, to 200 mg/mL, resulted in lower gold loading of the sutures. This was most likely due to a higher coagulation rate of AuNPs at 200 mg/mL, which decreased the effective AuNP loading. We did observe a greater amount of coagulation/precipitation at the end of the 24 h incubation in the 200 mg/mL solution than in the 50 and 75 mg/mL solutions. In the more crowded 200 mg/mL AuNP solution, the chance of individual AuNPs colliding and coagulating/precipitating out of the solution phase would be higher. This had no apparent effect on the micro-CT signal, however. Loading of AuNPs at all of the concentrations tested introduced radiopacity to PPDO sutures, as compared to the untreated controls, on both micro-CT and x-ray. Since all AuNP solutions yielded similar radiopacity, we propose that, of the infusion solution concentrations tested, the 50 mg/mL is optimal. These results proved the feasibility of infusing PPDO sutures with AuNPs to produce a radiopaque resorbable medical device.

A part from radiopacity, mechanical properties such as durability, flexibility, and radial strength are also critical for biomedical applications^6^. The strength of the material is of critical importance to the efficacy of an IVC filter, since a material that is too weak could lead to device failure or VTE. To test whether insertion of foreign particles disrupted the intrinsic scaffold structure and weakened the mechanical strength of the PPDO fibers, we first examined the tensile strength of the AuNP-infused sutures. We found no significant difference in tensile strength of the untreated or infused sutures, suggesting that AuNP infusion did not change their mechanical strength. This was supported by measurement of the T_*m*_ of the AuNP-infused and untreated sutures. A change in T_*m*_ indicates a change in the degree of crystallinity of the polymer and thus in its mechanical strength^22, 23^. However, all measured T_*m*_ values peaked around 112 °C for both untreated and AuNP-infused sutures, indicating no change in crystallinity. Furthermore, neither particle size nor concentration of AuNPs affected the mechanical strength of the infused samples. In an earlier study, infusion of PPDO with TIBA caused no significant decrease in T_*m*_, while infusion of PPDO with IBC (15 mg/mL) decreased T_*m*_ by 5 °C^12^. These results demonstrate that the mechanical strength of PPDO is better maintained by AuNPs than by iodine-based contrast agents.

Finally, we examined the cytotoxicity of the AuNP-infusing solutions *in vitro* to evaluate the biocompatibility of the AuNP-infused sutures. Because in the intended use, the AuNP-IVC filter will be in contact with normal cells, especially blood vessel cells, two normal cell lines – immortalized human vascular endothelial cell RF24 and lung fibroblast MRC5 cell lines – were used. Previous reports from other groups have demonstrated that AuNPs were nontoxic as a contrast agent^13, 21^. Our results support those published results.

On the basis of these results, we studied the long-term ability of PPDO to retain the AuNP within the polymer. AuNP-infused and untreated sutures incubated in PBS at 37 °C for up to 10 weeks were evaluated each week for radiopacity on micro-CT imaging and gold content by ICP-OES. No significant loss of radiopacity or gold content was observed over the 10-week evaluation period. This is important for the intended application because the amount of AuNPs in the filter must remain stable for visualization by micro-CT or x-ray. The intended dwell time of the infused IVC filters is 5 weeks, the same as untreated PPDO IVC filters, to capture thrombi and provide necessary protection for patients at risk for VTE. Hence it is important to maintain a stable gold content in the PPDO filters for at least 5 weeks to allow monitoring of the position and the integrity of the sutures. The degradation of PPDO monofilaments is caused by hydrolysis of the ester bonds in the material^22^. In the 10-week observation study, the PPDO monofilaments maintained their weight until week 7, suggesting that degradation via loss of low molecular weight chain segments from the suture does not happen until after week 7^22^. We showed that, despite hydrolysis of the PPDO and loss of chain segments, AuNPs were still embedded in the remaining PPDO fibers.

Additional preclinical evaluation is still needed, including biodistribution and toxicity studies of the infused AuNP during and after intended dwell time of the resorbable IVC filter. A porcine study is currently underway to determine not only the efficacy of the AuNP-infused PPDO in imaging and trapping clots but also its potential local and systemic toxicity.

## Conclusion

PPDO sutures were infused with AuNP and characterized. Our results suggest that infusion of AuNPs could enhance the radiopacity of PPDO while preserving the suture’s mechanical strength and integrity. Optimizing the AuNP concentration in the infusion solution could enhance radiopacity. AuNP-induced radiopacity could facilitate easy monitoring of the resorbable IVC filter, increasing its safety and efficacy.

## Materials and Methods

### Chemicals and Materials

PDS II PPDO suture (violet monofilament, Z880G) was purchased from Ethicon, Inc. (Somerville, NJ). Size 1 and size 2–0 were used. Dichloromethane (DCM, ACS reagent, ≥99.5%), toluene (ACS reagent, ≥99.9%), diethyl acetate (ACS reagent, ≥99.5%), gold (III) chloride trihydrate (HAuCl_4_·3H_2_O, ≥99.9%), tetrabutylammonium bromide (TBAB, ACS reagent, ≥98.0%), 1-octanethiol (≥98.5%), and sodium borohydride (NaBH_4_, ≥99%) were obtained from Sigma-Aldrich (St. Louis, MO). Gold nanoparticles (2 and 4 nm) were purchased from Nanocomposix (San Diego, CA). All chemicals were used without further purification unless otherwise noted.

### Synthesis of Gold Nanoparticles

The synthesis of 4-nm AuNP followed a well-known protocol^24, 25^. Briefly, a calculated amount of HAuCl_4_·3H_2_O was added to a mixture of distilled water and toluene with TBAB and allowed to react for 30 min. Then, 1-octanethiol was added and allowed to react for 1 h. NaBH_4_ was then added and allowed to react for another 30 min. At the end of the reaction, the toluene phase was separated and dried on a rotary evaporator, yielding the AuNPs. The AuNPs were resuspended in DCM for further analysis and infusion into the PPDO sutures. The ultraviolet–visible (UV-Vis) profile of AuNPs was monitored on a Cary 60 UV–Vis spectrophotometer (Agilent Technologies, Santa Clara, CA). Particle size was determined on a JEOL 1230 high contrast transmission electron microscope (TEM; JEOL USA, Inc., Peabody, MA) equipped with a digital camera.

### Infusion of PPDO with AuNPs

Sixty-centimeter sections of PPDO sutures were soaked in the AuNP-DCM solution for 24 h, air-dried for 24 h, then vacuum-dried for another 24 h. After drying, the AuNP-infused PPDO sutures were gently washed with ethyl acetate to remove any AuNPs clinging to the surface and vacuum-dried at room temperature for another 24 h. Two kinds of control sutures were prepared – DCM (no AuNP) soaked PPDO sutures and untreated PPDO sutures.

### Scanning Electron Microscopy

The morphologies of the treated PPDO sutures were examined by scanning electron microscopy (SEM). All suture samples were deposited on a conductive tape. A Nova NanoSEM 230 SEM (FEI, Hillsboro, OR) equipped with an EDX detector (the Octane Silicon Drift Detector (SDD), EDAX Inc., Mahwah, NJ) was used in Fig. 2B and C. All imaging (working distance of 5 mm, acceleration 5–10 kV) was done at room temperature and in a high vacuum (5E-6 Torr). SEM samples were coated with 3 nm Pt/Pd on a 208HR High Resolution Sputter Coater (Ted Pella, Inc., Redding, CA) and the images were obtained at a spot size 3. SEM-EDX samples were uncoated and the images were obtained at a spot size 4.

### Elemental Analysis

The total gold loading in the PDDO sutures was determined by using a Varian 720ES inductively coupled optical emission spectrophotometer (ICP-OES; Agilent Technologies) housed in the Department of Nanomedicine at Houston Methodist Research Institute. Briefly, AuNP-infused sutures were digested in aqua regia (concentrated nitric acid:hydrochloric acid 1:3, v/v) at 55 °C until completely dissolved. The resulting solutions were diluted in deionized water and the concentration of gold quantified on the ICP-OES. The experiments were conducted in triplicate for a single suture sample. Data were analyzed using the regression results obtained from calibration curves (R^2^ ≥ 0.998) plotted between the concentrations of the standard gold solutions and absorbance of gold at 191.896, 197.742, 208.207, 211.068, 242.794, and 267.594 nm^26^.

### Tensile Strength

The tensile strength of the AuNP-loaded and control suture samples was assessed by using the eXpert 7601 tensile testing machine with MTESTQuattro software (ADMET, Norwood, MA). Each sample was taken from the container containing the infused suture samples, where the suture was positioned with the knot pointed midway between both arms to allow for consistency in force distribution relative to the knot. The tensile strength of the suture samples was assessed at a cross-head speed of 25 cm/min. Each specimen was stretched to failure; the maximum load was recorded as weight (kg) and tabulated for analysis.

### Thermal Properties

The thermal properties (glass-transition temperature, melting enthalpy, and melting temperature [T_*m*_]) of the AuNP-infused and control PPDO monofilaments were obtained via differential scanning calorimetry (DSC). The thermal history of the AuNP-infused and control sutures was assessed on a Thermal Analysis SDT Q600 (TA Instruments, New Castle, DE) at a scanning speed of 10 °C/min in an argon atmosphere. All samples weighed ~100 mg and were placed in platinum pans.

### Micro-Computed Tomography Imaging

The radiopacity of the AuNP-infused and control PPDO filters was determined by using a CT-eXplore Locus RS preclinical *in vivo* scanner (GE Medical Systems, London, ON, Canada). This system uses a tungsten source x-ray tube operating at 80 kV and 450 mA. The x-ray source and charge-coupled device (CCD)-based detector gantry revolve around the subject in roughly 1.0-degree increments. The “MedRes-Mimic10 min-Mimic” protocol was used and ran for approximately 10 min. Uncoated filters and solvent-dipped sutures were used as controls. The radiopacity of the materials in HU was quantified by an in-house software application.

### X-ray Imaging

An MX-20 digital cabinet x-ray (Faxitron Bioptics, Tucson, AZ), a high-throughout planar system, was used for x-ray imaging. The MX-20 model has an energy range of 10 to 35 kV and a tube current of 300 μA. The focal spot size is <20 μm and the x-ray beam angle is 40 degrees. The resolution of the system is up to 50 lp/mm (10 lp/mm contact mode). The exposure time can be adjusted from 1 s to 19 s. Samples were run at an exposure time of 19 s at 25 kV.

### Cytotoxicity

The cytotoxicity of the AuNPs was examined in immortalized human vascular endothelial cell RF24 cells and normal lung fibroblast MRC5. Cells were cultured in Eagle’s minimum Essential Medium (EMEM) and minimum essential media (MEM) completed with 10% fetal bovine serum (FBS) and 1% penicillin and streptomycin, respectively, and maintained at 37 °C in a 5% CO_2_ atmosphere. Sixty centimeters of AuNP-infused sutures were soaked in 5 mL of cell culture medium for 24 h. The resulting extracts were used for the cytotoxicity evaluation. Cells were seeded (2000 per well) on a 96-well plate and allowed to grow for 24 h. The cells were then treated with the extracts, dilutions of the extracts, or cell culture medium with no extracts (control) for another 72 h. At the end of this period, the cell culture medium containing the extract was removed from each well. Phenol red-free cell culture medium with 3-(4,5-dimethylthiazol-2-yl)-5-(3-carboxymethonyphenol)-2-(4-sulfophenyl)-2H-tetrazolium (MTS) was added to each well, and cells were allowed to react for another 3 h. At the end of this period, UV absorbance at 490 and 690 nm was read, and the (O.D._490 nm_–O.D._690 nm_) was used to quantify cell viability.

### Long-term Radiopacity and Gold Content of AuNP-infused PPDO

Thirty-three PDS II size 2–0 PPDO sutures were individually infused with 4-nm AuNP in a solution containing 50 mg/mL of gold. Another 33 untreated sutures were used as controls. All sutures were immersed in 0.1 M phosphate-buffered saline solution (PBS, pH = 7.4) and incubated at 37 °C for up to 10 weeks. Three sutures from each group were collected each week; their gold content was quantified by ICP-OES and their radiopacity by micro-CT.

### Statistical Analysis

One-way analysis of variance (ANOVA) followed by a post hoc test (*p* < 0.05) was used to evaluate the statistical significance of differences between treatment groups in tensile strength and cytotoxicity. *T*-test was used to compare the infusion between 4- and 2-nm AuNPs.
